# Precise phenotyping method using image data for carcass marbling score in Hanwoo cattle

**DOI:** 10.1371/journal.pone.0318058

**Published:** 2025-01-24

**Authors:** Wang Yeol Lee, Phuong Thanh N. Dinh, Yoonji Chung, Hyo-Jun Lee, Yeong Jun Koh, Hyun Joo Kim, Shil Jin, Jaeho Lee, Jun Heon Lee, Ki Yong Chung, Seung Hwan Lee, Hyung-Yong Kim

**Affiliations:** 1 Korea Institute for Animal Products Quality Evaluation, Sejong, Republic of Korea; 2 Department of Bio-AI Convergence, Chungnam National University, Daejeon, Republic of Korea; 3 Division of Animal & Dairy Science, Chungnam National University, Daejeon, Republic of Korea; 4 Hanwoo Research Institute, National Institute of Animal Science, Pyeongchang, Republic of Korea; 5 Department of Beef Science, Korea National College of Agriculture and Fisheries, Jeonju, Republic of Korea; 6 Insilicogen, Inc., Yongin, Republic of Korea; South China Agricultural University, CHINA

## Abstract

With the development of the Korean economy, demand for high-quality beef, specifically Hanwoo beef, is escalating, with marbling traits—measured by the widely used marbling score—being a key contributor to meat palatability. The differences between the high-quality and the lower-quality meat, according to the satisfaction of the customers, are not the result from only the degree of marbling but also from the delicacy of the marbling flecks distribution. Using the computer marbling analysis technique, an index for quantifying marbling fineness of 256 sirloin cuts at 12^th^– 13^th^ thoracic vertebra named F7 index was developed in this study. F7 index is defined as the standard deviation of the ratios of marbling particles area to the tile area, was developed in this study. At the optimal step size of 70 tiles per axis, F7 index discriminated dramatically the finely marbled and coarsely marbled sirloin with Beef Marbling Score from 6 to 9, with *P* value = 1.340 × 10^−27^ < 0.05 of. Although the efficiency of computer image analysis procedure with the F7 index is still being optimized, the F7 index shows great potential to enhance the accuracy of Hanwoo beef quality grading alongside marbling score and support the development of improved breeding strategies for the Hanwoo cattle population in Korea.

## Introduction

Meat is widely recognized as an essential component in human well-balanced human diet as a valuable source of protein, iron, zinc, selenium, and vitamin B12 [1]. The consumption of meat, especially beef, has seen an upward trend with the continuous development of the economy [2]. In Korea, Hanwoo cattle, originally bred as draft animals, have evolved into a high-quality beef source with notable marbling levels, and the consumption demand is escalating in recent years [3]. From 2012 to 2022, beef consumption in Korea increased by 57.4%, with domestically supplied consumption accounting for 37.68% and imports making up 62.32% in 2022 [4]. Recently, the emphasis of the Korean breeding strategy has been placed not only on quantity but also on quality of the beef by applying National Hanwoo Breeding system and artificial insemination [5].

In general, the quality of beef has become a significant factor influencing consumption patterns and prices [6]. Several studies highlight that consumer choices hinge largely on tenderness, juiciness, flavour, and the degree of marbling [7, 8]. Marbling, defined as the presence of white fat flecks (intramuscular fat—IMF) in the muscle, are the most widely used determinants for estimating the meat quality [9]. In Korea, beef carcass grading system consists of Quality Grade (QG) with 5 levels (1++, 1+, 1, 2, and 3) and Yield Grade (YG) with 3 level (A, B, and C) [5]. The Beef Marbling Standard system with 9 grades called the Beef Marbling Scores (BMS; 1 = devoid, 9 = very abundant) [10], detecting the abundance level of IMF, has been used as one of the factors for Quality Grade of Hanwoo. In this system, achieving QG 1++ requires BMS scores of 8 or 9, QG 1+ requires scores of 6 or 7, QG 1 requires scores of 4 or 5, QG 2 requires scores of 2 or 3, and QG 3 requires a score of 1 [11]. Moreover, an alternative beef grading system with 9 grades is widely used in the US [12]; while Japanese professional graders use 12-level grading system for evaluating beef marbling [13]; another 10-level grading system is also used in Australia [14].

An alternative perspective showed that the marbling score grading may be overrated [15]. Since marbling scores are assessed subjectively by grading experts in USA, Japan, Australia, and Korea [16], causing the inconsistency [17]. Thus, a need to have more objective methods has given the establishment of an instrumental appraisal. The development of computer technology has given rise to the application of computer vision to meat evaluation, which was first introduced in this field in 1989. The computer image analysis (CIA) technique processes the meat photos through computer algorithms to extract key characteristics [16]. It offers stability and greater speed in comparison to human visual assessment [18]. For example, the development of adaptive algorithms for automatic segmentation of the beef longissimus dorsi muscle and marbling, using edge detection and region-growing techniques to delineate marbling features accurately, has enabled more precise identification and analysis of marbling patterns, offering improved accuracy and reliability over traditional methods [19]. Additionally, Yoshikawa et al. used discriminant threshold selection method and run length processing to enhance Japanese beef grading consistency and objectivity [6].

The use of computer vision and image analysis not only enhances the objectivity and precision of marbling evaluations but also addresses consumer preferences and market demands for specific marbling characteristics. Interestingly, while consumers generally prefer highly marbled meat, they have recently tended to avoid meat with excessive amounts amount of marbling, although it still needs to be tender [2, 5]. Furthermore, the customers do not prefer the meat with large marbling flecks (coarse meat) [15, 20], which cannot be clearly cannot be clearly differentiated from its counterpart using marbling score grading. To address these challenges, a complement indicator of marbling trait was established–the marbling fineness. Marbling fineness is a term to describe how delicately the intramuscular fat flecks distribute within the meat, which could be used to precisely describe the fat accumulation and allocation. There have been some previous studies developing marbling fineness indices, mainly in Japan, such as fineness index of Kuchida [21]. However, the application of this index on Hanwoo cattle was not successful [15] due to several differences, including the difference in body frame size that Wagyu cattle is big while Hanwoo cattle have a medium one; and the cutting position of Wagyu is 6^th^ - 7^th^ *thoracic vertebrae* but that of Hanwoo is 12^th^ - 13^th^. This is the major motivation to develop a fineness index that is more applicable to the domestic Hanwoo in Korea. In this study, a more suitable evaluation indicator, named F7 index, for measuring marbling fineness using computer image analysis on image data of Hanwoo beef would be introduced.

## Material and methods

### Muscle samples and image information

A total of 256 longissimus thoracis muscle samples at13^th^ *thoracic vertebrae* were taken from 256 Hanwoo cattle at 30 months old [11]. The meat samples including 3 levels of marbling distribution—coarse, medium, and fine marbled meat, ranging from BMS grade 6 to grade 9 (Table 1) were used in this study. The assessments of the carcasses were conducted by experts from the Korea Institute for Animal Products Quality Evaluation (KAPE) in 2018. The KAPE experts were responsible for assigning scores to the carcasses and determining the fat distribution in each cross-section, categorizing them as fine, medium, or coarse marbling based on their expertise and established evaluation criteria. Fine marbling meat has the most evenly distributed fat across the cross-section while coarse one has irregular fat distribution with larger fat streaks. Isometric images of the sirloin cross-section were photographed using HK-333 photography equipment [21] at KAPE. Guidelines for animal health and welfare from the Animal Care and Use Committee (NIAS) were followed (Ethics Committee Approval Number: 2015–150). The number of images used for the image analysis process is as in Table 1.

**Table 1:** Number of sirloin cross-section images provided in this study.

Marbling fineness	BMS 6	BMS 7	BMS 8	BMS 9	Total
Coarse	20	20	20	22	82
Medium	20	21	24	22	87
Fine	25	22	20	20	87
Total	65	63	64	64	256

Grade 6–7 are classified as 1+. Grade 8–9 are classified as 1++.

### Image analysis for marbling fineness

**Fig 1:**
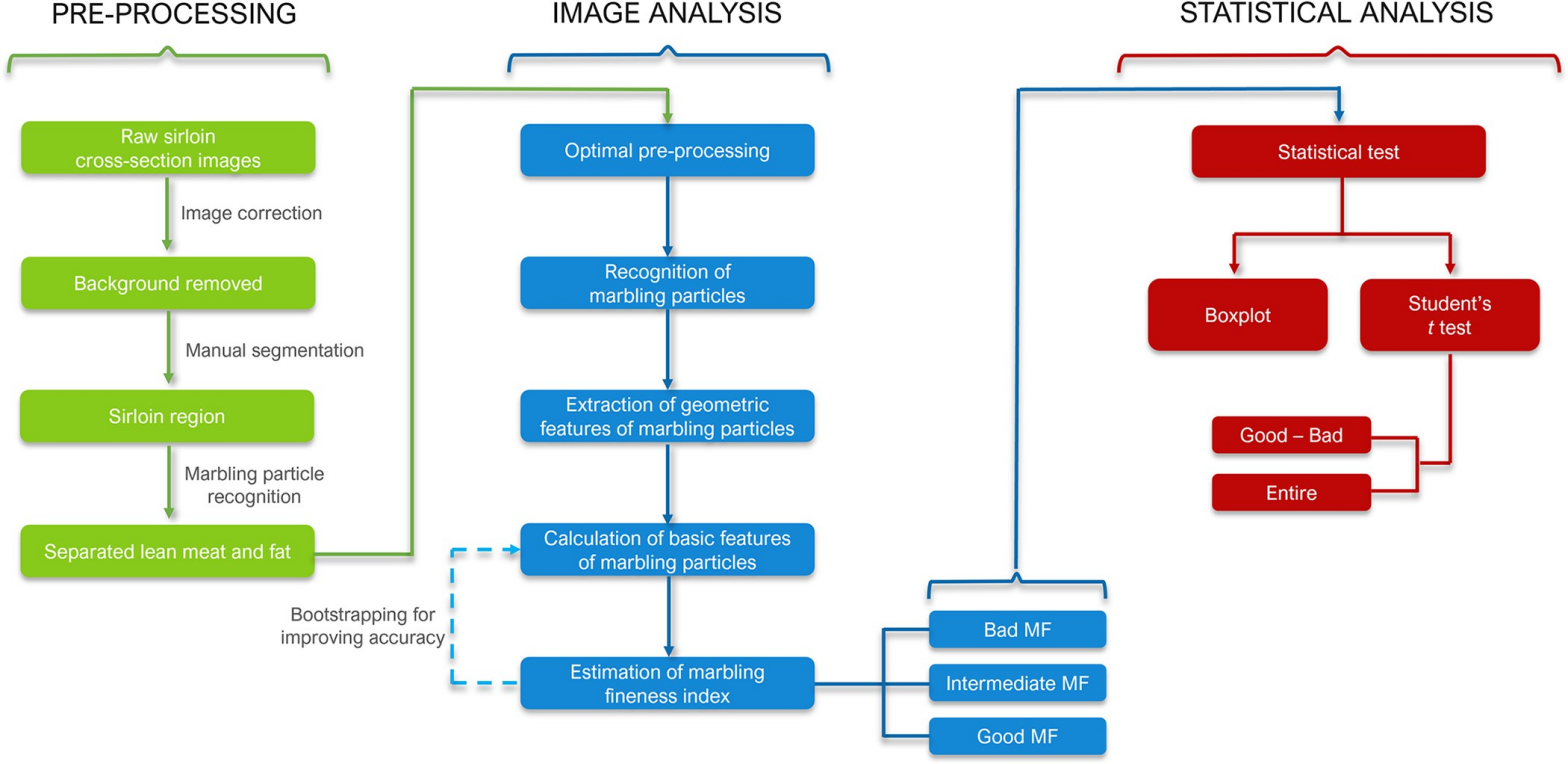
Summary of marbling image data analysis process.

**Fig 2:**
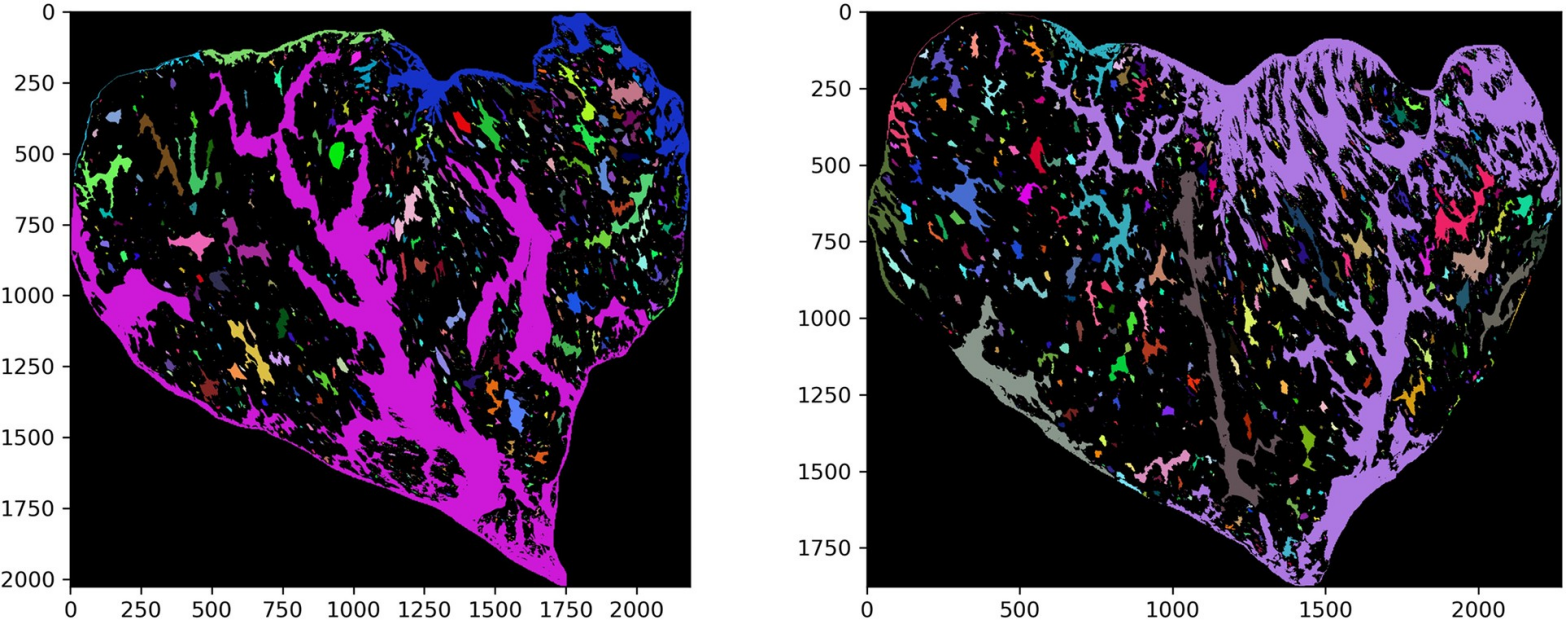
The recognized marbling particles of the BMS 9 coarse marbling image (left) and the fine marbling image (right).

The entire analysis process for the provided images is summarized in Fig 1, including 3 main steps. First, image pre-processing, which comprises the optimization for recognizing marbling particles and segmentation of the marbled particle areas, was performed. The sirloin part of the meat image was manually selected by drawing the corresponding boundary lines using Photoshop. Background was removed. Consequently, the marbling particles—the white parts in the image—were segmented from the lean muscle regions—the red parts, turning the images into binary ones. Threshold were detected for this segmentation and the algorithm that distinguishes these binary pixel values is called image thresholding. As the most effective and widely used threshold technique using high-value filter, Otsu’s thresholding method was adopted in this study, detecting a customized threshold for each of the images [22]. After the partitioning of lean muscle and fat, the marbling particles were obtained with the removal of meat regions for analysis (Fig 2). Third, the geometric characteristics of the marbling particles, including: shape, length, area, and perimeter were extracted, and the number of particles is also pulled out, where a_i_ is the area of i^th^ particle (sorted by area in ascending order, unit: pixel), n is the total number of particles, and p_i_ is the perimeter of i^th^ particle (sorted by perimeter in ascending order, unit: pixel).

$$A_{total}=\sum_{i=0}^{n}a_{i}$$

$$P_{total}=\sum_{i=0}^{n}p_{i}$$

A_50_: area of i^th^ particle when $\frac{\sum_{i=0}^{n}a_{i}}{A_{total}}=0.5$

P_50_: perimeter of i^th^ particle when $\frac{\sum_{i=0}^{n}a_{i}}{A_{total}}=0.5$

Based on this, marbling fineness estimation indices for arbitrary sirloin cross-section images were developed. In this study, we did 11 test functions in total to find out which one is the best indicator for the marbling fineness (Table 2). Among these, the F1a, F1b, F2a, F2b, F7, and F8 were the candidates.

**Table 2:** List of marbling fineness index candidates.

Indicator	Description	Formula
F1a	Number of marbling particles	n
F1b	Total of marbling particle area	A_total_
F2a	Area of i^th^ particle at 50% cumulation of marbling particle area	A_50_
F2b	Area of i^th^ particle at 50% cumulation of marbling particle area to total area	A_50_/A_total_
F3a	Perimeter of i^th^ particle at 50% cumulation of marbling particle perimeter	P_50_
F3b	Perimeter of i^th^ particle at 50% cumulation of marbling particle perimeter to total perimeter	P_50_/P_total_
F4	The number of marbling particles that smaller than the P50	n when $\frac{\sum_{i=0}^{n}a_{i}}{A_{total}}$ < 0.5
F5	Average of squared perimeter of marbling particle to area ratio	Avg(P^2^/A)
F6	Area of i^th^ particle at 50% cumulation of marbling particle area multiplied by its perimeter	A_50_*P_50_
F7	Standard deviation of tile marbling area to tile area ratios	SD(A_marbling_ /A_tile_)
F8	F2b to F7 ratio	F2b/F7

Unit of area and perimeter is pixel.

F1a index is the number of marbling particles, and F1b was total of marbling particle area in pixel, which was defined as A_total_. Marbling particles were sorted ascendingly based on their area. At the cumulating level of 50%, the differences of these particles between the fine and coarse meat appeared to be the most conspicuous (S3 Fig). F2 index was developed taking advantage of this observation. F2a is the area of the i^th^ particle (sorted in ascending order) in pixel when the cumulation of area from the 1^st^ to the i^th^ particle reach 50% of the total marbling area in the sirloin cross-section; and F2b is the ratio between F2a and total marbling area to correct the absolute length differences among images. The equations of F2 indices can be written as:

$$F2a=A_{50}$$

$$F2b=\frac{A_{50}}{A_{total}}$$

**Fig 3:**
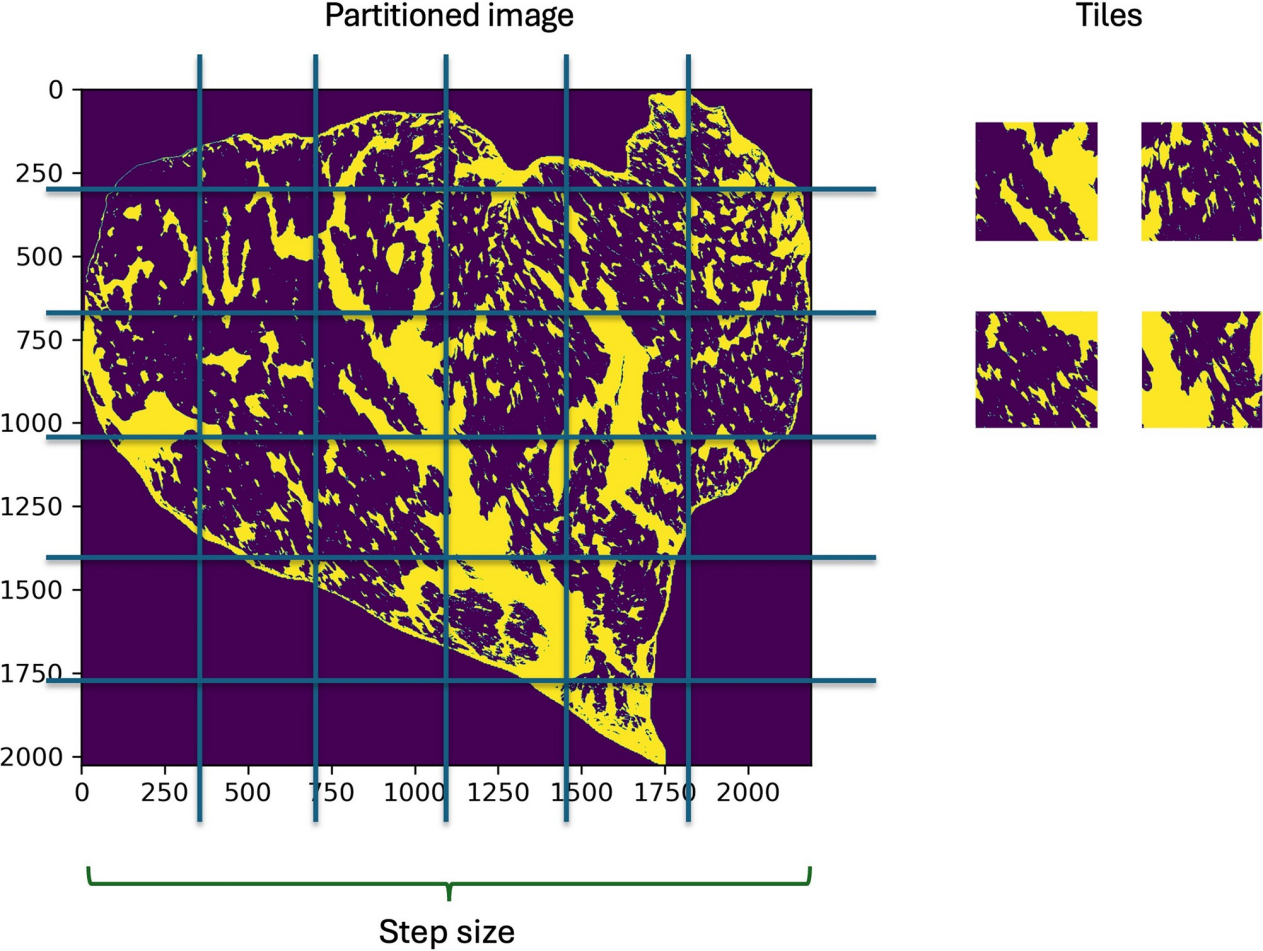
Image partitioning illustration for F7 marbling fineness index estimation.

To express the “fineness” term, the distribution of marbling particles should be delicate over the cross-section. The F7 method provides a more suitable way to detect this delicacy by partitioning the image into smaller fragments. The process can be conducted in three steps:

(1) Divide the image into smaller parts, each part is called a tile. The number of tiles along x-axis equals to that along y-axis and is defined as the step size (Fig 3).

(2) Select the grids located in the meat region to find the ratio of the area of marbled particles to the tile area.

(3) Apply the standard deviation of the area ratios.

The equation of the F7 index can be performed as:

$$F7=\sqrt{\frac{\sum_{i=1}^{n}{\left(x_{i}-\bar{x}\right)}^{2}}{n-1}}$$

Where n is the number of tiles of the image, *x_i_* is the ratio of the area of marbling particles in the tile i to the total area of that tile, and $\bar{x}$ is the mean of *x_i_*. The lower the F7 index, the better the marbling fineness. The step size was optimized. A range of step sizes from 5 to 95 tiles per axis was used for estimating F7 marbling fineness index. The step size where F7 index can clearly distinguish the fine marbling group and coarse marbling group of BMS grade 9 was chosen. And finally, F8 index was developed as the ratio of F2b and F7 indices, with the equation can be written as:

$$F8=\frac{F2b}{F7}$$

Finally, the certainty of the indices was improved by bootstrapping.

### Statistical analysis

In this study, three groups of marbling fineness—fine, medium, and coarse—using the indices were compared. t tests were used to compare the fine and coarse groups, while ANOVA was applied to evaluate all three groups, where fixed terms included the marbling fineness groups. After being quantified, the distribution of the indices will be visualized by boxplot, and the index is detected to be statistically significant under the threshold of *P* value < 0.05.

## Results

The of marbling indices revealed significant distinctions in marbling fineness across different BMS grades. The F1a index (total number of marbling particles) was significantly higher in fine marbled meat compared to coarse marbled meat for BMS grades 6, 7, and 8 (*P* value < 0.05). However, for BMS grade 9, the difference was not significant (*P* value = 0.123 > 0.05). The F1b index (total area of marbling particles) increased with higher BMS grades, but it did not differentiate well between fine and coarse marbled meat for grades 7 and 8 with *P* value = 0.514 and 0.569 > 0.05, respectively. Similarly, the F2a and F2b indices did not distinguish between fine and coarse marbling for any of the BMS grades, as all tests returned insignificant *P* values (*P* value = 0.596 and 0.933, respectively).

**Fig 4:**
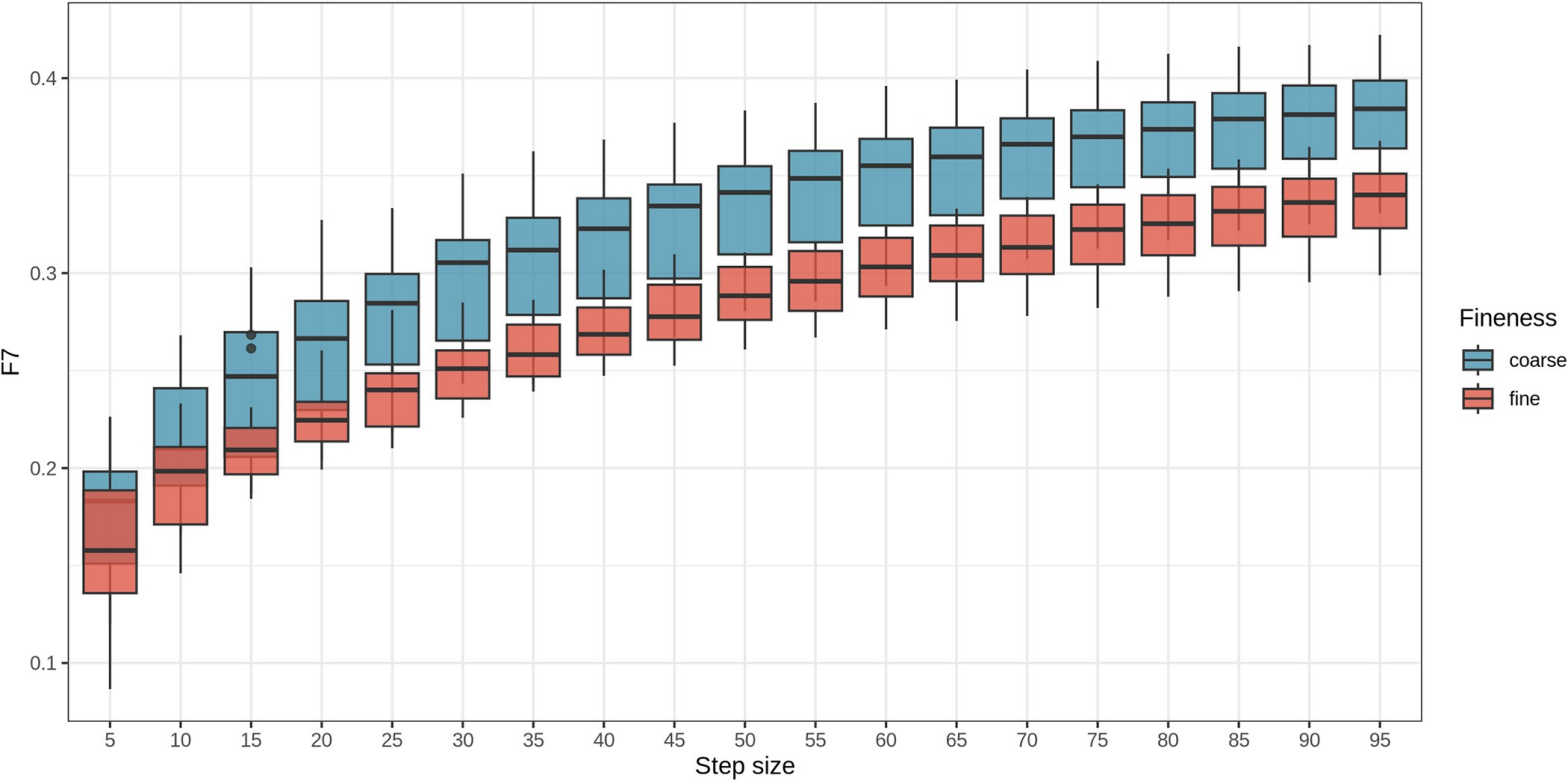
Boxplots for comparison between BMS 9 coarse marbling group and fine marbling group of F7 marbling fineness index by step sizes.

**Fig 5:**
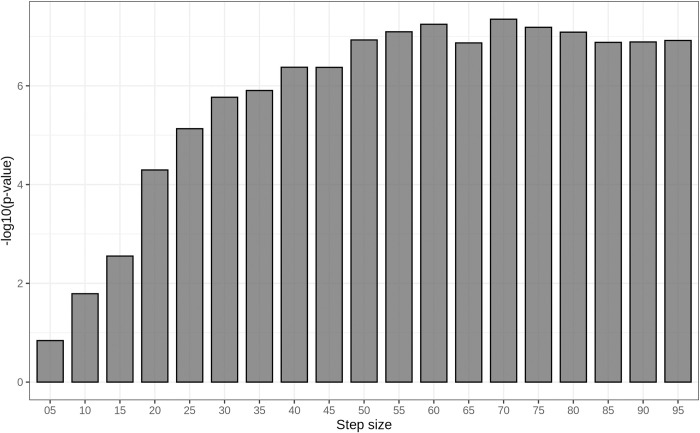
Significance level of the separation of coarse and fine BMS-9 beef images using F7 index at step sizes from 5 to 95 tiles per image axis.

**Fig 6:**
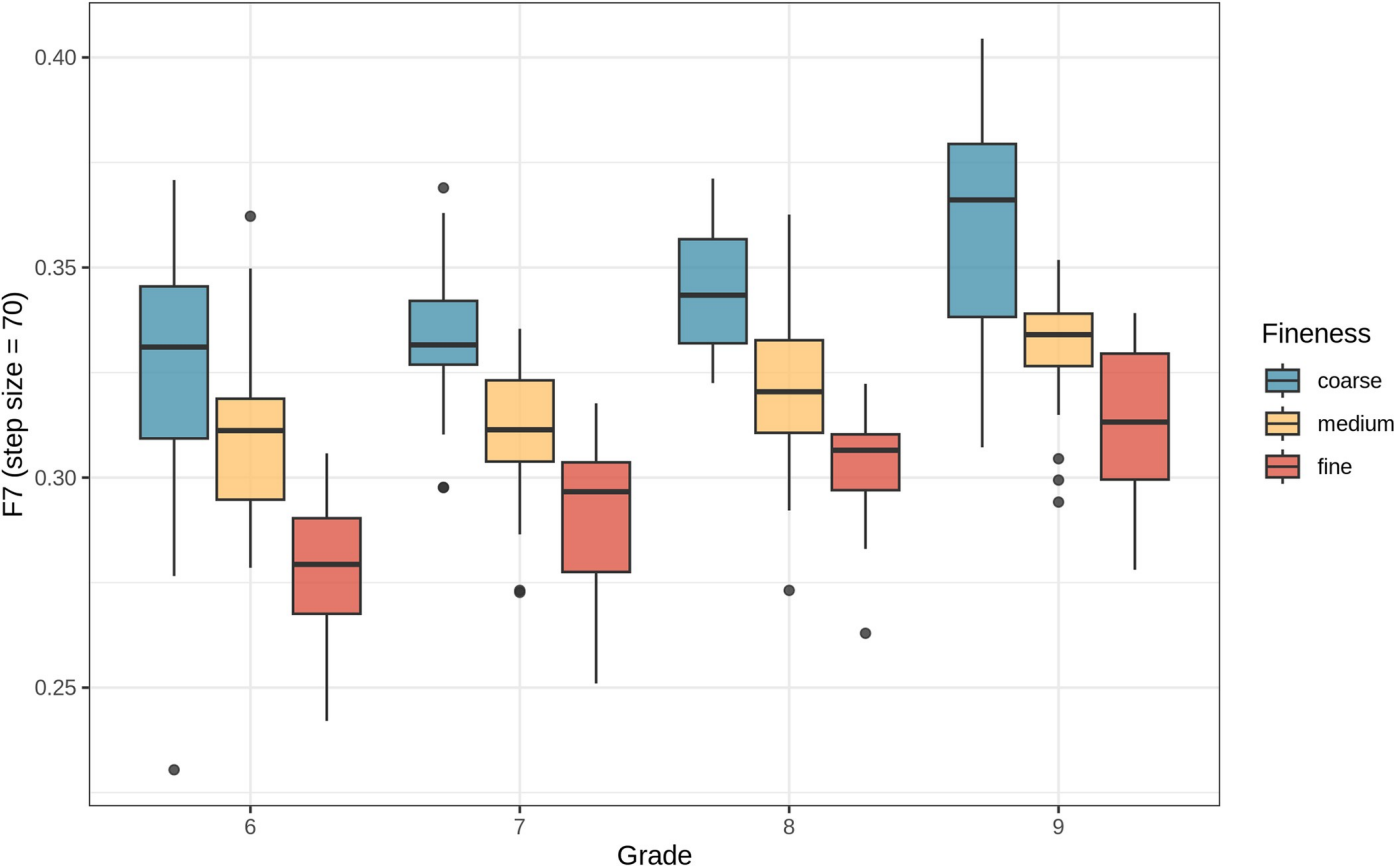
Distribution of F7 marbling fineness index at step size = 70 of 4 BMS grades.

Fig 4 shows the differences between the two groups as the step size increases. In contrast, the F7 index demonstrated the clearest distinction between fine and coarse marbling across all BMS grades. At a step size of 70 tiles per axis (70 × 70 tiles per image), the separation between the two groups was highly significant (*P* value = 3.6 × 10^*−19*^ <0.05) (Fig 5), establishing this step size as optimal for F7 index estimation. The distribution of the F7 index at step size 70 is presented in Fig 6 and described in Table 3. Overall, the F7 index between fine and coarse marbling was significant for all grades with the *P* value of the total test was 1.340 × 10^−27^ < 0.05.

**Table 3:** Statistics and ANOVA results of F7 marbling fineness index at step size = 70 between groups of 4 grades.

	Coarse mean	Medium mean	Fine mean	*P* value
BMS 6	0.323	0.310	0.277	9.782 × 10^−08^
BMS 7	0.333	0.312	0.292	4.690 × 10^−09^
BMS 8	0.345	0.321	0.303	1.667 × 10^−09^
BMS 9	0.360	0.330	0.313	2.307 × 10^−09^
Total	0.341	0.318	0.295	1.340 × 10^−27^

Finally, the F8 index did not show substantial differences between coarse and fine marbled meat for any grade except BMS 9, with a general *P* value = 0.807 > 0.05. Detailed results of the F1a, F1b, F2a, F2b, and F8 indices can be found in the Supplementary.

## Discussion

In the beef industry, marbling traits, particularly the marbling fineness trait, which has been emphasized recently, are considerably influential in meat quality appraisal. A variety of instrumental methods were developed to evaluate them more precisely. In image analysis, especially for meat with high marbling score, segmentation is an important step to determine the boundaries between different parts of the meat, significantly affecting to the evaluation accuracy. In the study of Adi et al. [23], Otsu’s thresholding method, which is a nonparametric discriminant threshold selection method, was used for marbling segmentation, achieving 90% accuracy in classifying beef images by marbling score using a decision tree. Similarly, Shiranita et al. used this technique in their study [19] and obtained up to a 100% of correct rate with a custom feature called “normalized run length run number vectors.” Therefore, Otsu’s thresholding method was applied in our study to detect marbling particles as one of the most effective as one of the most effective ways to maximize the variance between meat and marbling (S2 Fig). However, this success required strict standards for input images and the manual pre-adjustment of the images, including the background removal and size normalization. Kuchida also mentioned that this method could give misleading binary conversions due to the uneven brightness, which could make the grayscale histogram inconsistent [17]. This problem remains the main drawback of this pipeline when the preprocessing of the sirloin cross-section image is not automated, resulting in the low performance of the entire process. Further studies are needed to address this issue, with clustering-based methods—independent of the histogram—offering a potential solution.

After segmentation, primary features such as the number of marbling particles or the area of marbling are commonly usually used for marbling score evaluation. For instance, Shiranita et al. used features including the percentage of marbling, number of large marbling particles, number of small particles, total number of particles, and the amount of scatter of the distribution of particles in the binarized region, to estimate marbling score of meat [19]. As mentioned earlier, the marbling score alone recently cannot fully capture the customer demand for beef marbling. Unlike some reported research aiming to evaluate beef using the original marbling score, our study formulated marbling fineness indices to quantify marbling information as numerical values based on the characteristics of marbling particles in sirloin images. The F1a and F1b indices using the total number of marbling particles and the total marbling particle area have been part of the features in visual appraisal by human graders for years in Japan [24] and Korea [9]. Although these indices were computed by the computer—offering greater consistency—they still could not effectively distinguish the fineness of marbling in our study. The total area of fat was also used as an indicator of marbling score by McDonald and Chen finding a correlation of only 0.47 with sensory marbling scores [20]. These results indicate that while the total marbling area increases with higher grades, this measure alone is unreliable and does not describe the distribution of marbling particles, which results in coarse meat due to the presence of large marbling flecks.

Among the marbling fineness indices analysed in this study, the F7 index best differentiated between fine and coarse marbling groups. The F7 index is calculated as the standard deviation of the ratio of marbling area to step size ratios of tiles. The concept of creating ratios to estimate the distribution of IMF in meat is not new. For example, Kuchida et al. developed a marbling fineness index in 2006 for Wagyu cattle in Japan. This fineness index is calculated by dividing the number of small marbling particles, ranging in size of 0.01 to 0.5 cm^2^, by the area of the loin-eye [21] which was shown to be highly correlated with the Japanese Marbling Standard (12 grades), with correlation r^2^ = 0.47 [25]. This technique was also suggested to have potential for measuring marbling fineness of longissimus muscle [26]. However, the approach of partitioning the image to evaluate the even distribution by applying standard deviation appears to better capture the "fineness" concept. The partitioning provides information on IMF distribution, while standard deviation shows the variation in IMF distribution across tiles. Therefore, combining that information into the F7 index results in more accurate assessment of marbling fineness, where a higher F7 index value indicates coarser marbling. We found that at the step size of 70 tiles per axis, F7 index significantly separated the coarse and fine marbling meat images with BMS 6 to 9 in Hanwoo cattle. The outcome also revealed that overall, the F7 index of fine marbling is lower than that of coarse marbling, as expected from its definition. F7 index is considerably linear correlated with Kuchida’s fineness index [21] in negative direction, with Pearson’s correlation = -0.854 (S8 Fig) since it measures the difference of IMF content between tiles instead of summing the amount of fat content. The F7 index is intended as an additional indicator for grading in the beef market. Although F2b and F7 were found to be the most promising indicators for distinguishing between fine and coarse marbling, further research on the combination of F2 and F7—resulting in the F8 index—may also be meaningful. However, the results from this combination were contrary to expectations. Currently, our goal is to identify a more suitable, effective, and resource-efficient indicator for computer image analysis.

In conclusion, the need to quantify marbling fineness, which plays a crucial role in assessing the quality of marbled meat, has been increasingly emphasized. This study introduces a development of the corresponding marbling fineness index–the F7 index–to improve the accuracy of meat appraisal using image information. A significant distinction between coarse and fine marbled sirloin cross-sections was demonstrated using the F7 index as an indicator for computer image analysis, which helps enhance the quality evaluation system of Hanwoo beef and contributes to future breeding strategies.

## Supporting information

S1 Table Statistics and ANOVA results of F1a marbling fineness index between groups of 4 grades. (DOCX)

S2 Table Statistics and ANOVA results of F1b marbling fineness index between groups of 4 grades. (DOCX)

S3 Table Statistics and ANOVA results of F2a marbling fineness index between groups of 4 grades. (DOCX)

S4 Table Statistics and ANOVA results of F2b marbling fineness index between groups of 4 grades. (DOCX)

S5 Table Statistics and ANOVA results of F8 marbling fineness index between groups of 4 grades. (DOCX)

S1 Fig Marbling particle recognition changes according to the threshold value algorithm changes. (TIF)

S2 Fig Thresholding algorithms and their thresholding efficiency. (TIF)

S3 Fig Cumulative distribution of marbling particle area of BMS grade 9 cross-section beef images. (TIF)

S4 Fig Distribution of F1a marbling fineness index at 4 BMS grades. (TIF)

S5 Fig Distribution of F1b marbling fineness index at 4 BMS grades. (TIF)

S6 Fig Distribution of F2a marbling fineness index at 4 BMS grades. (TIF)

S7 Fig Distribution of F2b marbling fineness index at 4 BMS grades. (TIF)

S8 Fig Pearson’s correlation between Kuchida’s marbling fineness index and F7 index. (TIF)

S9 Fig Distribution of F8 marbling fineness index at 4 BMS grades. (TIF)
