# Correction: Precise phenotyping method using image data for carcass marbling score in Hanwoo cattle

**DOI:** 10.1371/journal.pone.0323551

**Published:** 2025-05-05

**Authors:** Wang Yeol Lee, Phuong Thanh N. Dinh, Yoonji Chung, Hyo-Jun Lee, Yeong Jun Koh, Hyun Joo Kim, Shil Jin, Jaeho Lee, Jun Heon Lee, Ki Yong Chung, Seung Hwan Lee, Hyung-Yong Kim

The second affiliation of the first author is not indicated. Wang Yeol Lee is also affiliated with #3: Division of Animal & Dairy Science, Chungnam National University, Daejeon, Republic of Korea.
