# Supplementary material for: Precise phenotyping method using image data for carcass marbling score in Hanwoo cattle
Source: PLoS One. 2025 Jan 24;20(1):e0318058. doi: 10.1371/journal.pone.0318058 (PMC11760004; doi:10.1371/journal.pone.0318058)
Supplement: S1 Table — (DOCX) [file pone.0318058.s001.docx]

Table S1. **Statistics and ANOVA results of F1a marbling fineness index between groups of 4 grades**

|  | Coarse mean | Medium mean | Fine mean | *P* value |
| --- | --- | --- | --- | --- |
| BMS 6 | 1893.200 | 1817.850 | 2502.000 | 0.034 |
| BMS 7 | 1959.250 | 3067.143 | 3448.591 | 0.001 |
| BMS 8 | 2210.750 | 2587.583 | 3116.350 | 0.005 |
| BMS 9 | 2018.045 | 2179.182 | 2520.250 | 0.123 |
| Total | 2020.256 | 2423.115 | 2886.793 | 6.443 × 10^-07^ |

.
