# Supplementary material for: Precise phenotyping method using image data for carcass marbling score in Hanwoo cattle
Source: PLoS One. 2025 Jan 24;20(1):e0318058. doi: 10.1371/journal.pone.0318058 (PMC11760004; doi:10.1371/journal.pone.0318058)
Supplement: S2 Table — (DOCX) [file pone.0318058.s002.docx]

Table S2. **Statistics and ANOVA results of F1b marbling fineness index between groups of 4 grades**

|  | Coarse mean | Medium mean | Fine mean | *P* value |
| --- | --- | --- | --- | --- |
| BMS 6 | 554533.200 | 544789.100 | 454740.400 | 0.004 |
| BMS 7 | 632331.800 | 677042.600 | 641670.000 | 0.514 |
| BMS 8 | 745983.300 | 706958.700 | 729477.800 | 0.569 |
| BMS 9 | 1044835.000 | 897606.600 | 849952.000 | 0.008 |
| Total | 751748.000 | 710667.000 | 656021.200 | 0.014 |
