# Supplementary material for: Precise phenotyping method using image data for carcass marbling score in Hanwoo cattle
Source: PLoS One. 2025 Jan 24;20(1):e0318058. doi: 10.1371/journal.pone.0318058 (PMC11760004; doi:10.1371/journal.pone.0318058)
Supplement: S3 Table — (DOCX) [file pone.0318058.s003.docx]

Table S3. **Statistics and ANOVA results of F2a marbling fineness index between groups of 4 grades**

|  | Coarse mean | Medium mean | Fine mean | *P* value |
| --- | --- | --- | --- | --- |
| BMS 6 | 5.450 | 8.600 | 4.360 | 0.141 |
| BMS 7 | 6.800 | 4.047 | 4.091 | 0.200 |
| BMS 8 | 4.250 | 4.208 | 4.300 | 0.938 |
| BMS 9 | 4.545 | 4.864 | 5.950 | 0.305 |
| Total | 5.244 | 5.345 | 4.644 | 0.596 |
