# Supplementary material for: Precise phenotyping method using image data for carcass marbling score in Hanwoo cattle
Source: PLoS One. 2025 Jan 24;20(1):e0318058. doi: 10.1371/journal.pone.0318058 (PMC11760004; doi:10.1371/journal.pone.0318058)
Supplement: S4 Table — (DOCX) [file pone.0318058.s004.docx]

Table S4. **Statistics and ANOVA results of F2b marbling fineness index between groups of 4 grades**

|  | Coarse mean | Medium mean | Fine mean | *P* value |
| --- | --- | --- | --- | --- |
| BMS 6 | 0.003 | 0.003 | 0.006 | 0.308 |
| BMS 7 | 0.002 | 0.003 | 0.005 | 0.108 |
| BMS 8 | 0.002 | 0.002 | 0.004 | 0.807 |
| BMS 9 | 0.001 | 0.003 | 0.004 | 0.104 |
| Total | 0.003 | 0.003 | 0.005 | 0.933 |
