# Supplementary material for: Precise phenotyping method using image data for carcass marbling score in Hanwoo cattle
Source: PLoS One. 2025 Jan 24;20(1):e0318058. doi: 10.1371/journal.pone.0318058 (PMC11760004; doi:10.1371/journal.pone.0318058)
Supplement: S5 Table — (DOCX) [file pone.0318058.s005.docx]

Table S5. **Statistics and ANOVA results of F8 marbling fineness index between groups of 4 grades**

|  | Coarse mean | Medium mean | Fine mean | *P* value |
| --- | --- | --- | --- | --- |
| BMS 6 | 1.101 × 10^-05^ | 1.654 × 10^-05^ | 1.006 × 10^-05^ | 0.405 |
| BMS 7 | 1.205 × 10^-05^ | 6.103 × 10^-06^ | 6.648 × 10^-06^ | 0.170 |
| BMS 8 | 5.846 × 10^-06^ | 6.214 × 10^-06^ | 6.151 × 10^-06^ | 0.085 |
| BMS 9 | 4.530 × 10^-06^ | 5.526 × 10^-06^ | 8.155 × 10^-06^ | 0.038 |
| Total | 8.266 × 10^-06^ | 8.387 × 10^-06^ | 7.860 × 10^-06^ | 0.807 |
