# Supplementary figures and images for: Precise phenotyping method using image data for carcass marbling score in Hanwoo cattle
Source: PLoS One. 2025 Jan 24;20(1):e0318058. doi: 10.1371/journal.pone.0318058 (PMC11760004; doi:10.1371/journal.pone.0318058)

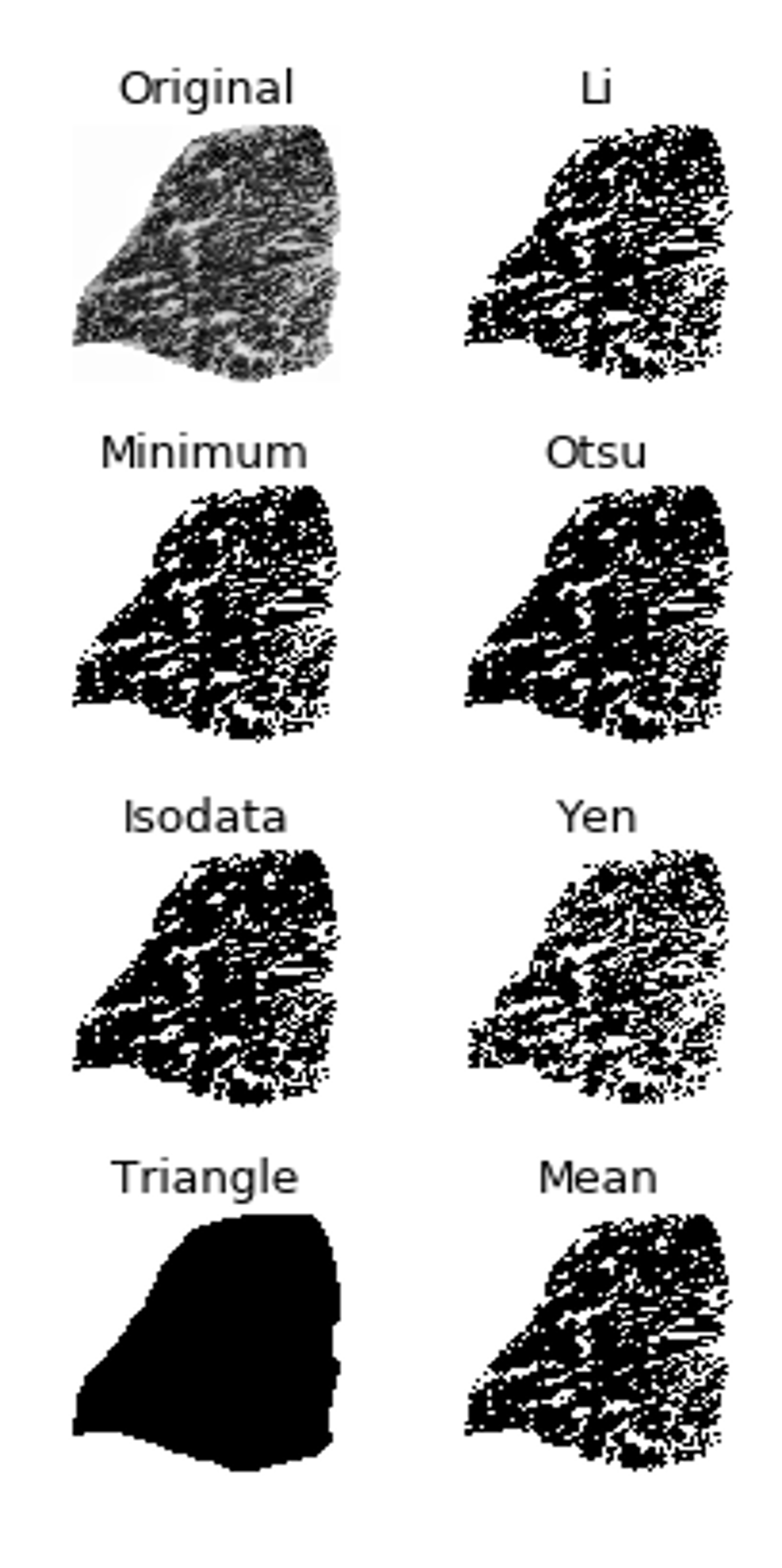

Supplement: S1 Fig — (TIF) [file pone.0318058.s006.tif]

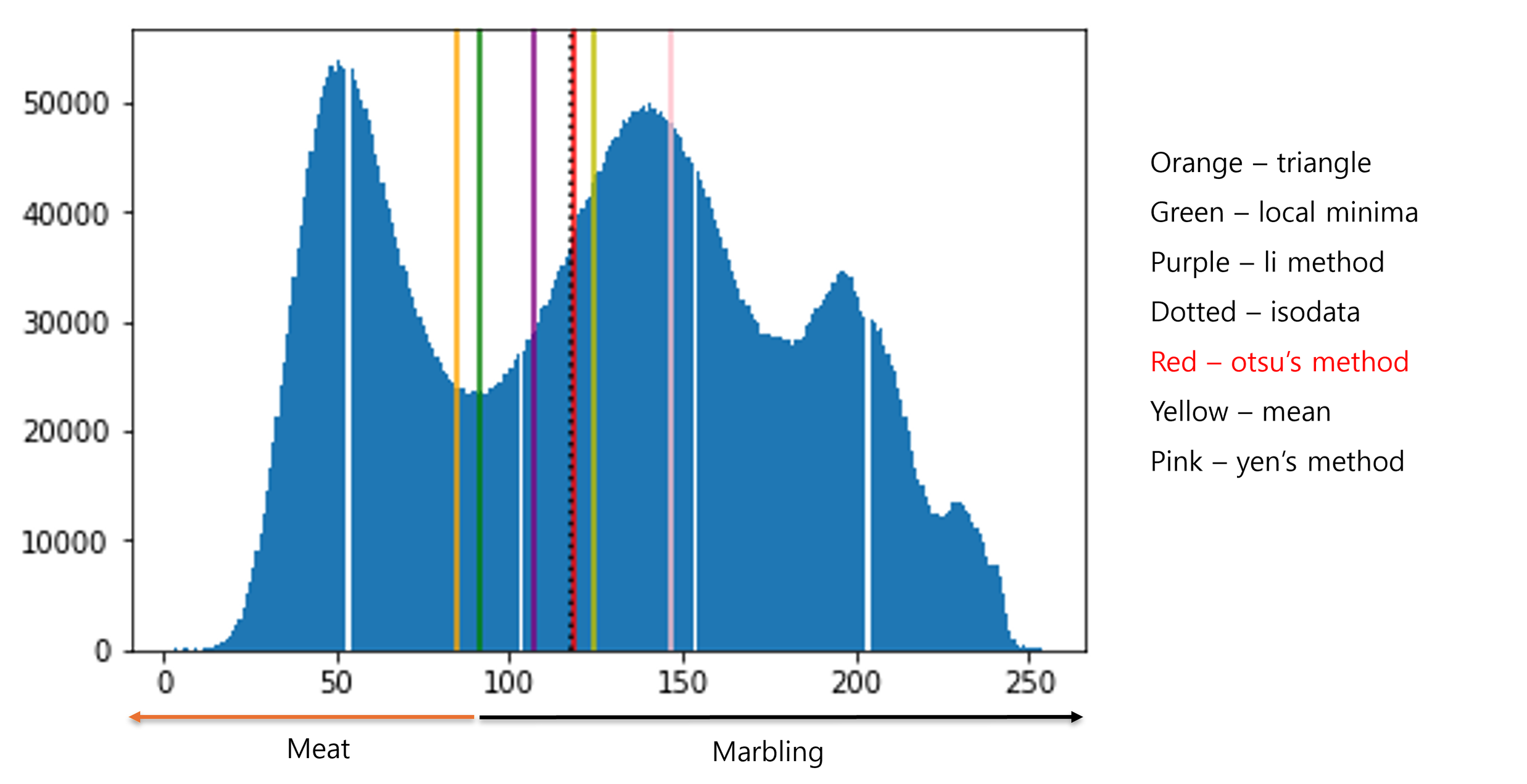

Supplement: S2 Fig — (TIF) [file pone.0318058.s007.tif]

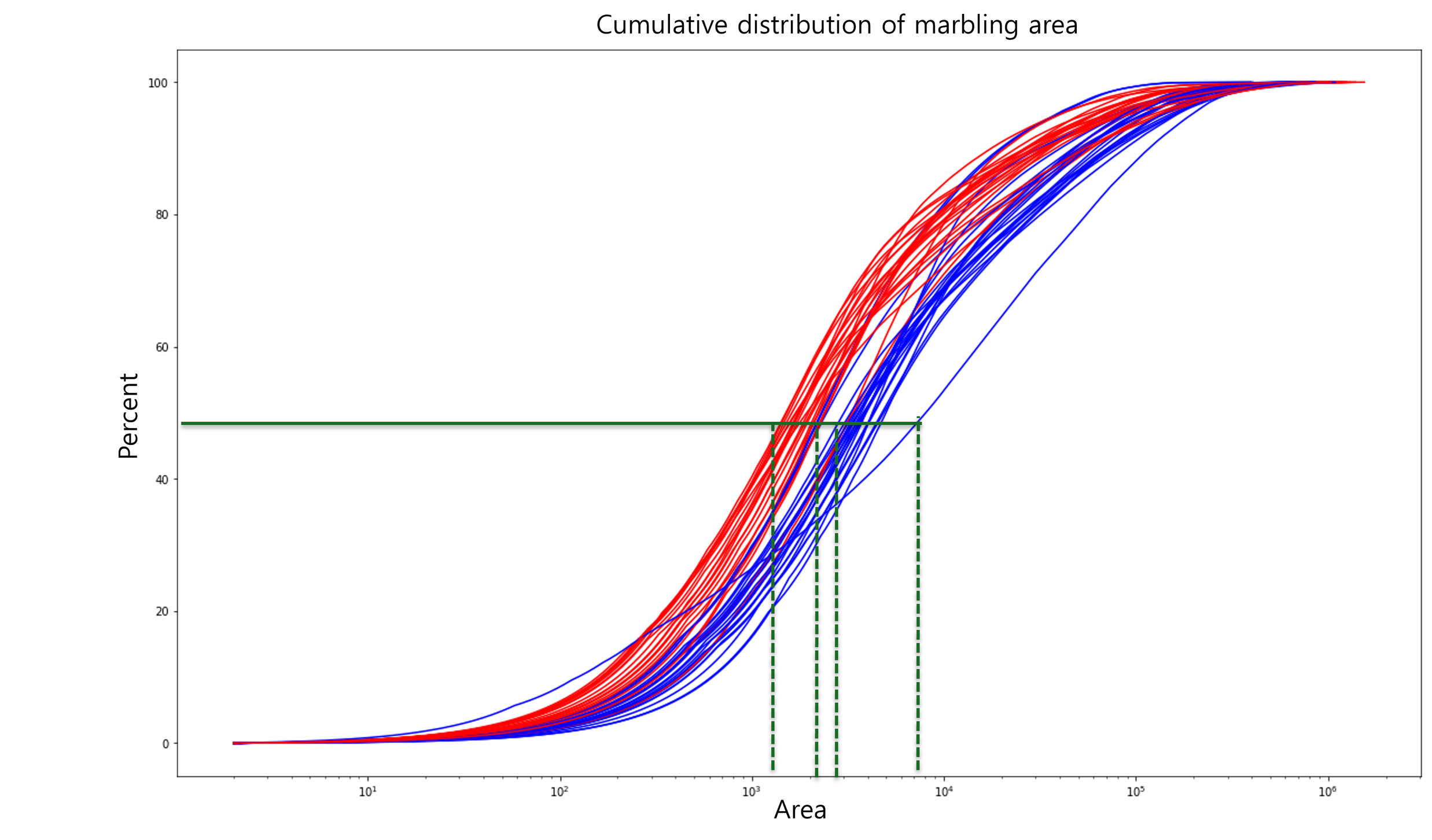

Supplement: S3 Fig — (TIF) [file pone.0318058.s008.tif]

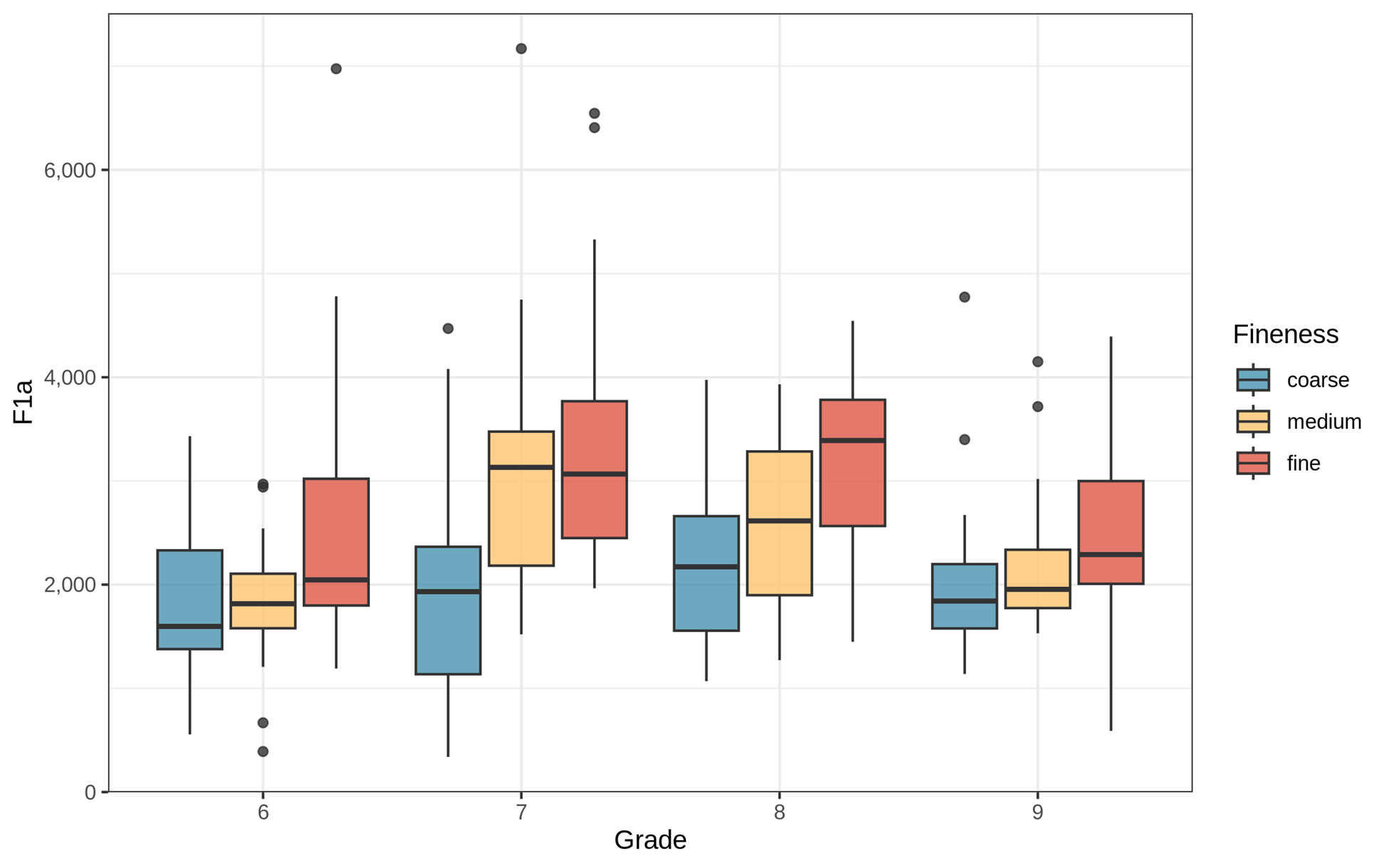

Supplement: S4 Fig — (TIF) [file pone.0318058.s009.tif]

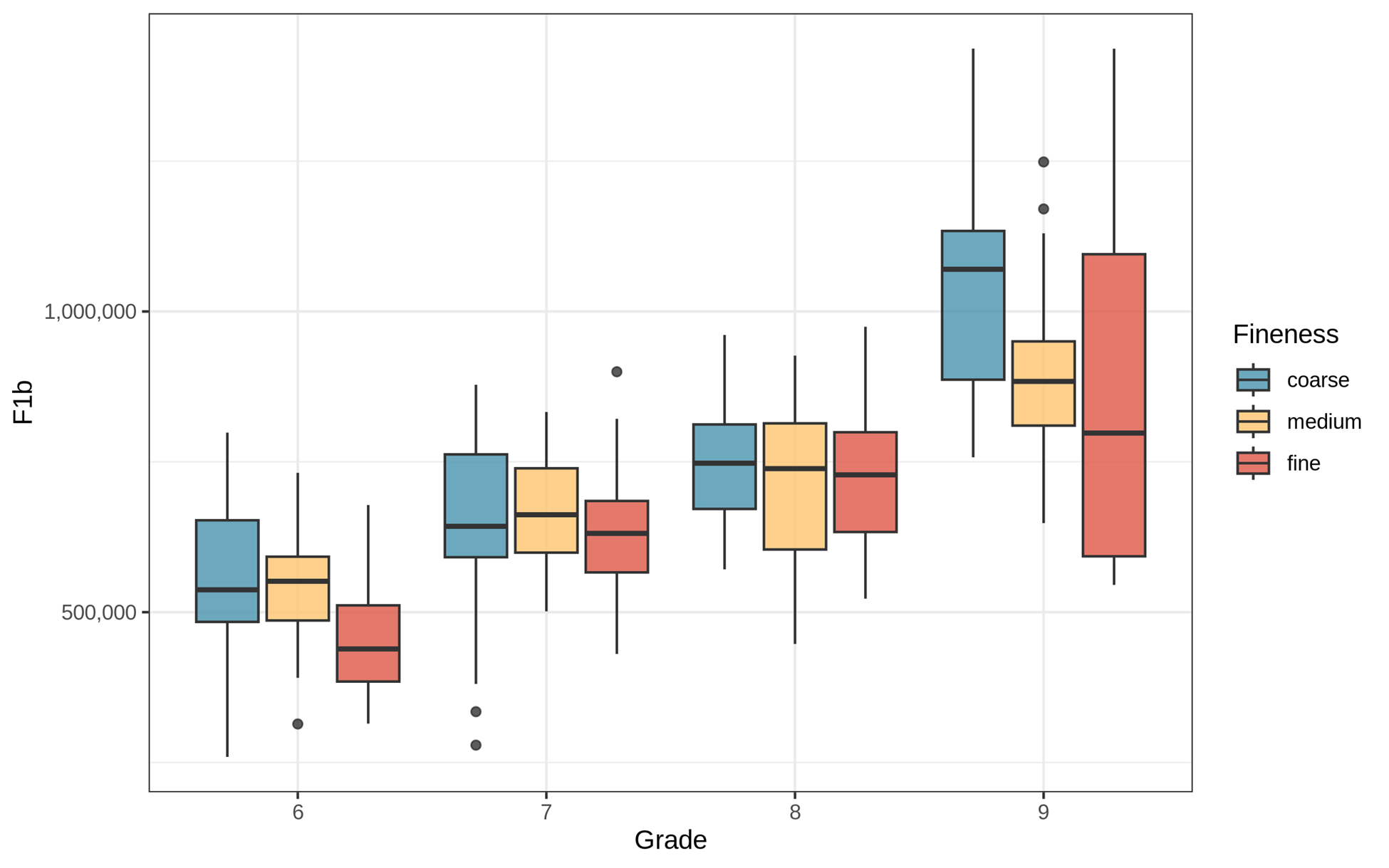

Supplement: S5 Fig — (TIF) [file pone.0318058.s010.tif]

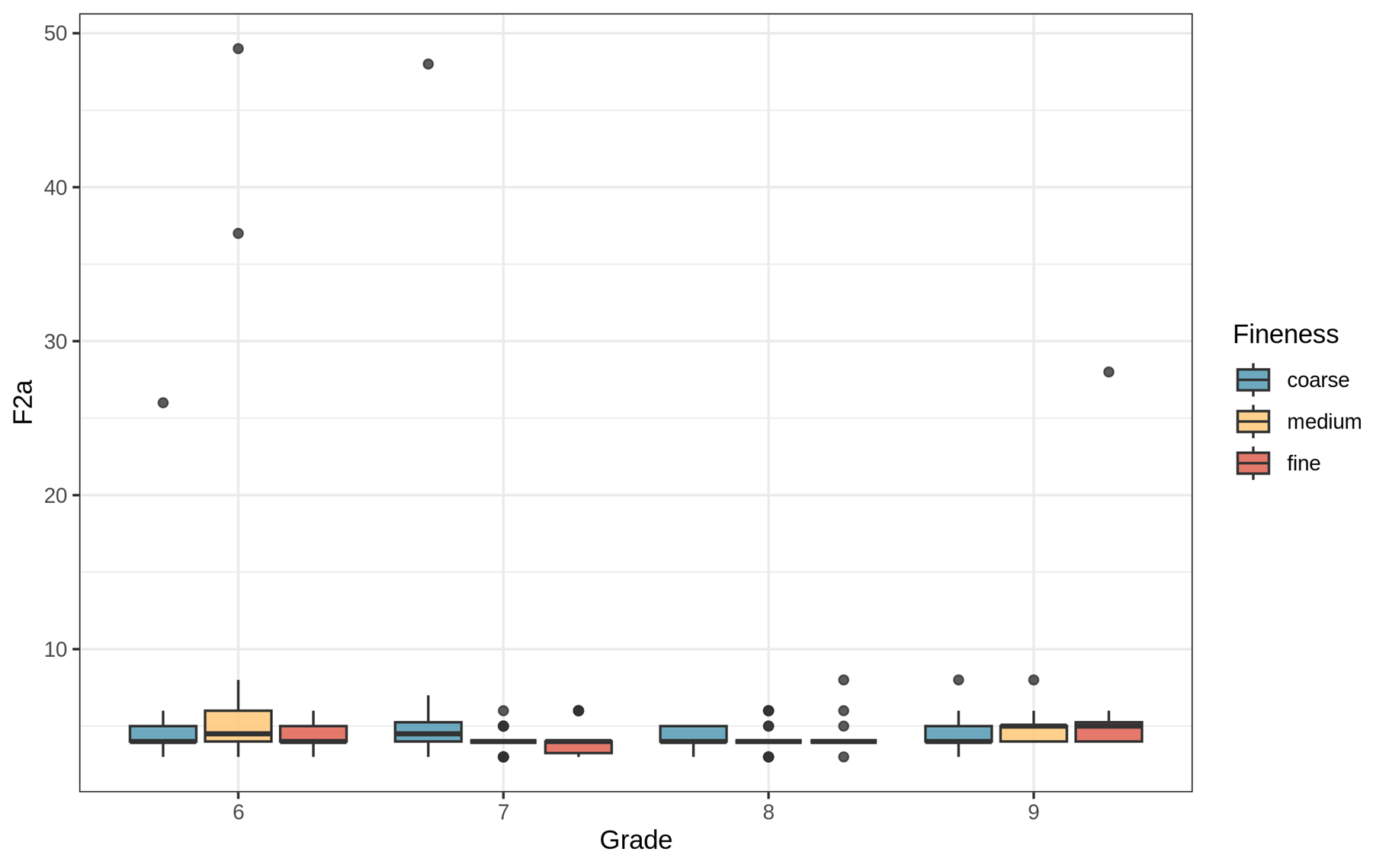

Supplement: S6 Fig — (TIF) [file pone.0318058.s011.tif]

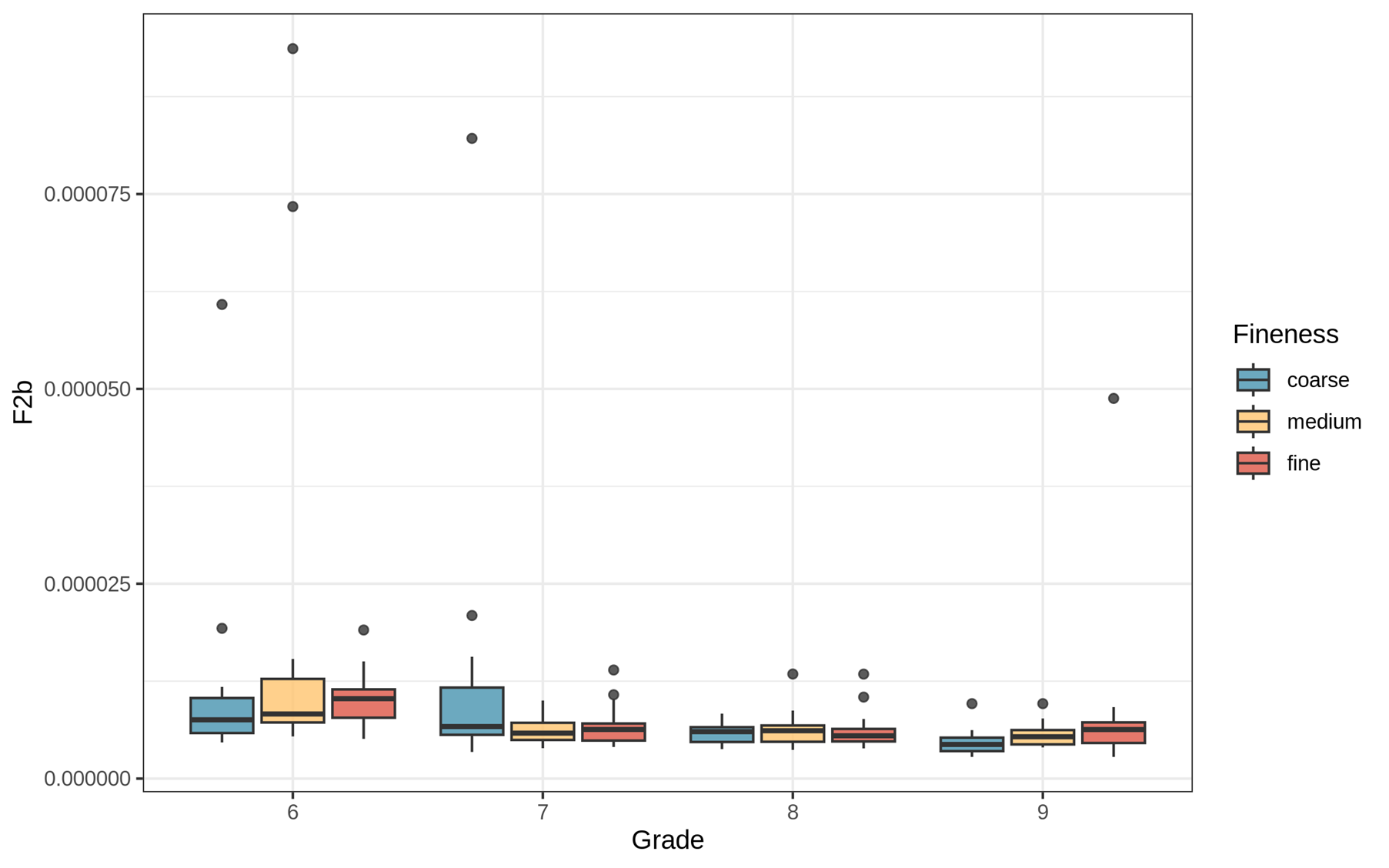

Supplement: S7 Fig — (TIF) [file pone.0318058.s012.tif]

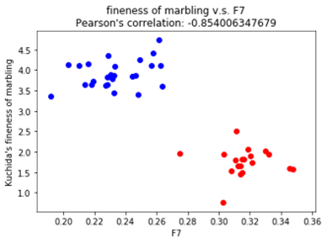

Supplement: S8 Fig — (TIF) [file pone.0318058.s013.tif]

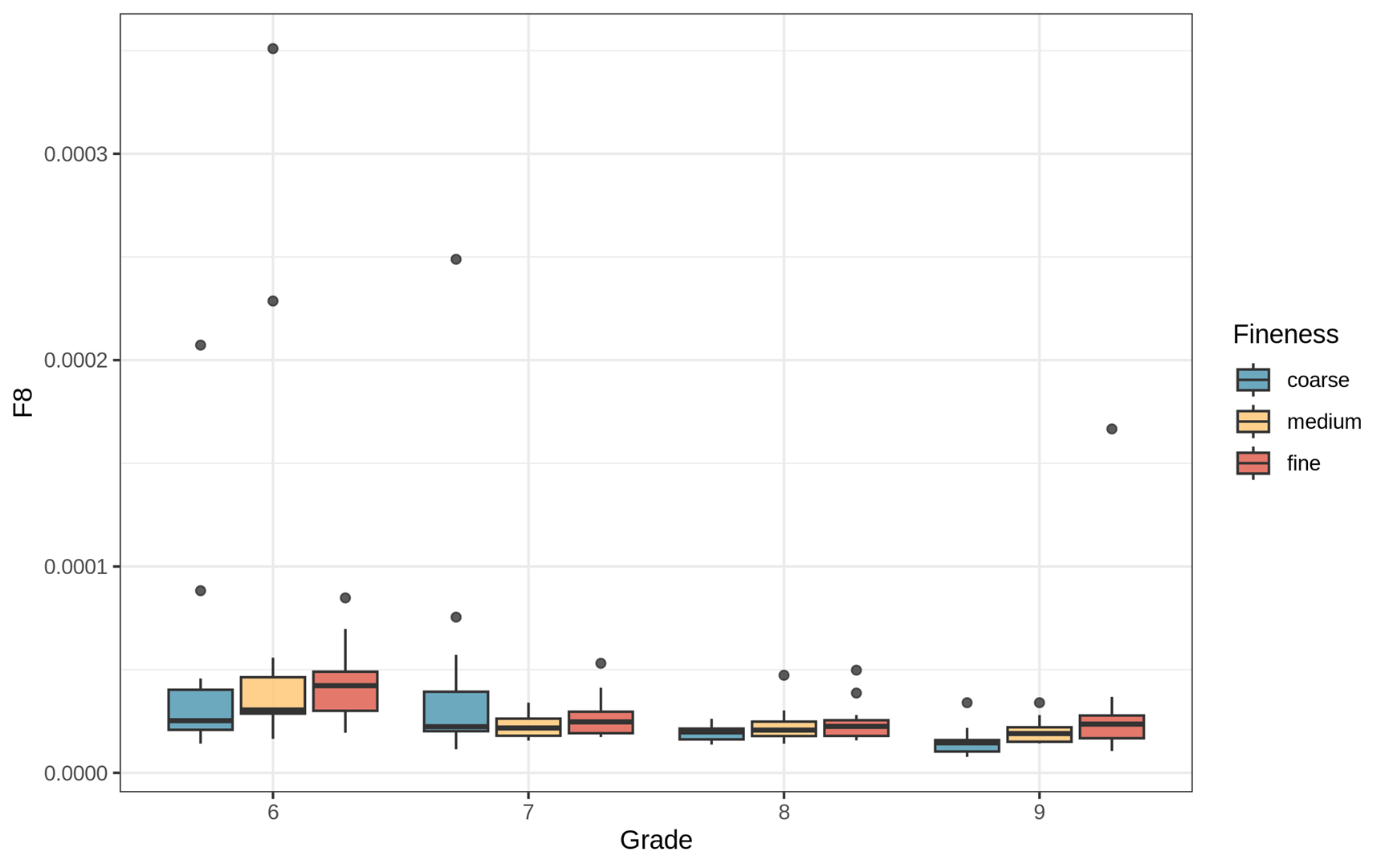

Supplement: S9 Fig — (TIF) [file pone.0318058.s014.tif]
